# From Genotype to Phenotype of Polish Patients with Pitt–Hopkins Syndrome concerning the Quality of Life and Family Functioning

**DOI:** 10.3390/jcm13092605

**Published:** 2024-04-29

**Authors:** Marlena Telenga, Anna Rozensztrauch, Kaja Giżewska-Kacprzak, Robert Śmigiel

**Affiliations:** 1 Department of Pediatrics, Endocrinology, Diabetology and Metabolic Diseases, Medical University of Wroclaw, 50-367 Wroclaw, Poland; 2 Department of Family and Pediatric Nursing, Wroclaw Medical University, 50-367 Wroclaw, Poland; 3 Department of Pediatric and Oncological Surgery, Urology and Hand Surgery, Pomeranian Medical University in Szczecin, 70-204 Szczecin, Poland

**Keywords:** Pitt–Hopkins syndrome, rare diseases, quality of life, disability

## Abstract

**Background**: Pitt–Hopkins syndrome (PTHS) is a rare genetic disorder affecting psychomotor, social, and intellectual development, caused by a mutation in the *TCF4* gene. The study aims to gather the phenotype and genotype data of PTHS patients from Poland and to assess the quality of life (QoL) and the impact of the disorders on the family. **Methods**: Eight families with PTHS participated in the study. To obtain data, the following standardized questionnaires were used: Questionnaire on Clinical Problems (QCP), the PedsQL™ Family Impact Module, and the QL-Disability Questionnaire. Additionally, a retrospective analysis of clinical examination, genetic consult, medical history, and genotype of each individual was performed. **Results**: All of the examined children exhibited a mutation in the *TCF4* gene and typical features of PTHS. The most prevalent clinical symptoms in the study group included typical PTHS appearance, intellectual disability (*n* = 5; as the rest of the patients were too young to be assessed), abnormal speech development (*n* = 8), reduced pain response (*n* = 7), constipation (*n* = 7), drooling (*n* = 7), cold extremities (*n* = 7), and disturbances in sensory integration processes (*n* = 7). The QL-Disability Questionnaire revealed a total QoL score of 67.7/100 for children with PTHS, while the QoL for their families in the PedsQL Family Impact Module was 53.82/100. The highest-rated domain was cognitive functioning (Median (Me) = 67.50; Standard Deviation (SD) = 21.95), while the lowest was daily activities (Me = 25.00; SD = 29.86). **Conclusions**: The study allowed the collection of data on the phenotype and genotype of children with PTHS living in Poland. Overall, our study showed that the QoL of children with PTHS is impaired.

## 1. Introduction

Pitt–Hopkins syndrome (PTHS; OMIM; 610954, ORPHA: 2896) is a rare genetic disorder characterized by severe neurodevelopmental, social, and intellectual impairment. PTHS was first described in 1978 by Australian doctors David Pitt and Ian Hopkins. However, in the 18th century, more than 200 years earlier, British painter William Kent immortalized a boy with a phenotype typical for PTHS in his painting entitled “Peter the Wild Boy”. According to British legends, this boy was found in the forest and never learned to speak [1,2].

The frequency of PTHS is not precisely determined due to the limited number of reported cases [3,4]. Estimates in the United Kingdom and the Netherlands range from 1 in 225,000 to 1 in 300,000 [1].

In 70% of children with PTHS, the syndrome is caused by a sporadic, de novo heterozygotic mutation of the *TCF4* gene, resulting in partial or total deletion. The remaining 30% of cases are attributed to de novo deletion on chromosome 18q21.1. To date, there is no significant genotype-to-phenotype correlation. However, variants in the first eight exons of the *TCF4* gene correspond with non-syndromic intellectual disability but not with the phenotype of PTHS [3,4,5,6,7,8].

Common symptoms include a lack or limitation of speech (less than 10% of patients use full sentences), motor developmental delay (only 30% of children aged 3 to 5 can walk without assistance), and disturbances of the autonomic nervous system (hyper-ventilation, apnea, constipation, gastroesophageal reflux disease, belching, drooling, dilated pupils, body temperature instability, urinary retention, peripheral blood flow disturbances). Other manifestations include seizures (40% of patients), vision impairments (50% of cases), stereotypical hand movements (approximately 60% of patients), and sleep disturbances (sudden sleep onset, difficulty falling asleep, parasomnias, and sleepwalking) [1,2,3,4,5,6,7,8,9,10,11,12].

A key diagnostic feature is facial dysmorphism, including bicoronal narrowing, narrow lateral portions of the eyebrows, widened nasal base/dorsum/tip/ala, prominent midface with full cheeks, wide mouth/full lips/upper lip with a prominent Cupid’s bow, and thickened earlobes [12]. Additionally, patients may exhibit microcephaly, seizures, wide lateral ventricles, narrow corpus callosum, inappropriate laughter, as well as slender and thin fingers [1,4,7,8,12]. According to the literature data, the clinical features of PTHS patients are distinctive enough to be distinguished from syndromes with similar features, such as Rett or Angelman syndrome [4].

Diagnostic recommendations published in 2019 guide genetic testing for PTHS, primarily involving *TCF4* gene sequencing and/or examination of microdeletions on 18q21 (microarray analysis) [1,12,13]. Due to the increased usage of next-generation sequencing (NGS) in rare disease diagnostics, there is a possibility of incidental detection of a *TCF4* gene variant in patients not initially suspected of PTHS. Therefore, a positive test result should be correlated with the patient’s phenotype according to the criteria in the presented recommendation [12].

While prenatal testing is suggested if PTHS is present in one of the previous children, it is not deemed necessary due to the low risk (2%) of the syndrome appearing in subsequent offspring. As there are no typical anatomical congenital anomalies in the PTHS picture a prenatal ultrasound examination alone cannot rule out the diagnosis and chorionic villus sampling and amniocentesis are recommended [4,12].

Currently, there is no targeted treatment for PTHS, and therapy focuses on symptomatic management. Nevertheless, the literature reports promising ongoing research toward the development of gene therapy [14,15]. Coordinated medical care involving collaboration among doctors, vision therapists, physiotherapists, and speech therapists is crucial to achieving maximum developmental milestones [12].

PTHS is a rare disease affecting various aspects of a child’s life and their family. It causes disability in both motor and intellectual aspects, leading to significant challenges for the entire family. The disease still requires further investigation, and the exact number of affected individuals in Poland is unknown. There have been no previous reports on the quality of life (QoL) of PTHS patients and their families.

This study aims to determine the genotype and phenotype of children with PTHS in Poland. Additionally, the research aims to assess the quality of life in the studied group and determine the impact of the disease on family functioning.

## 2. Materials and Methods

The study involved a group of 8 families of children with Pitt–Hopkins syndrome residing in Poland. The inclusion criteria for the study were a confirmed diagnosis of PTHS through genetic testing and an age below 18 years. Exclusion criteria included failure to respond to all questions in the questionnaires.

The group was gathered during recruitment in an online community uniting families with a diagnosis of PTHS and during a meeting for families of children with the syndrome, held on 10–12 June 2022, in Poland. There were 10 responses, and 8 families were included in the study. A retrospective analysis of clinical examination, genetic consult, medical history, and genotype of each individual was performed. Each participant (guardian) gave consent to participate in the study. Ethical approval for the research was obtained from the Local Bioethical Committee (Wroclaw, Poland).

The following questionnaires were utilized in the study:

a. Questionnaire on Clinical Problems (QCP) is a proprietary questionnaire concerning the clinical issues of children diagnosed with Pitt–Hopkins syndrome, collecting information about the family’s situation, pregnancy, childbirth, and subsequent stages of the child’s life, the patient’s phenotype and genotype, clinical problems of the child, organization of family life (treatment, rehabilitation, education, and functioning of the child and family in the context of the disease), and the impact of the COVID-19 pandemic on the functioning of the child and family.
b. The PedsQL 2.0 Family Impact Questionnaire consists of 8 modules with 36 items, rated on a scale from 0-never to 4-almost always. The score is then transformed into a scale from 0 to 100, where a higher score corresponds with better family functioning.
c. The QL-Disability Questionnaire in the version for caregivers, considers significant communication limitations in the children in the study group. It consists of 5 sections. Caregivers assessed the frequency of occurrence of specific behaviors and situations, which were then converted into points according to the questionnaire’s principles. The total score could range from 0 to 100, where a higher score corresponds with a better quality of life.

For statistical analysis, Statistica 13 software was used. For numerical results of the proprietary questionnaire and QoL scales, the mean, median, standard deviation, as well as maximum and minimum values, were calculated.

## 3. Results

### 3.1. Genotype of Children with Pitt–Hopkins Syndrome

The study involved eight families with one child diagnosed with PTHS each. Among the patients included in the study, there were two boys and six girls. The average age of the children was 7.25 years (Me = 6.5; SD = 3.85). The youngest child was 3 years old, and the oldest was 13 years old.

In each of the children included in the study, a mutation in the *TCF4* gene was identified. However, the gene variants harboring the mutation differed for each participant. All variants identified in patients had status de novo (none of the parents presented the variant identified in offspring). The description of abnormal *TCF4* gene variants is presented in Table 1.

Most results were obtained through targeted *TCF4* gene testing (sequencing)—four cases. In the remaining children, next-generation sequencing (NGS) with a mode of the dedicated panel was performed in two cases, or whole exome sequencing (WES) in two cases.

The age of the children at the time of diagnosis ranged from 2 to 8 years, average age was 4 years, with a median of 3 years.

### 3.2. Phenotype and Clinical Problems of Children with PTHS

Each of the examined children exhibited typical features of PTHS. Commonly present features included a broad nose (*n* = 7), full cheeks (*n* = 6), and a distinct Cupid’s bow (*n* = 6). Each child had a minimum of five characteristics typical of PTHS, fulfilling the first cardinal criterion for syndrome diagnosis. The occurrence of typical features in the study group is presented in Table 2.

In all examined children, caregivers reported speech development issues. Five of them use alternative communication methods, such as a book with pictograms (*n* = 3), a communication device (*n* = 2), gestures and facial expressions (*n* = 2), or the Makaton method (*n* = 1). Two children do not use any form of alternative communication. The main and typical neurodevelopmental problems of the examined children are illustrated in Table 3.

Concerning gastrointestinal disorders, commonly reported in the study group were widely spaced teeth (*n* = 7), constipation (*n* = 7), and apnea (*n* = 6). Issues with food intake, such as choking or gagging, did not require medical intervention in any of the children. During apnea episodes, no case of cyanosis was observed, and according to caregivers, the occurrence of apnea was not associated with seizures. Two cases of hyperventilation episodes were linked to strong emotions such as stress, fear, anger, or joy. Five out of eight examined children had frequent upper respiratory tract infections, and four had sporadic pneumonia. Respiratory infections in each child were treated with antibiotics more than three times, and two cases of pneumonia required hospitalization. Hirschsprung’s disease was not diagnosed in any of the examined children. Four children required daily medication for constipation. The prevalence of the most common gastrointestinal problems in the study group is shown in Table 4.

The most frequently observed orthopedic issues in children included foot deformities (flat feet, clubfoot, cavus foot). The study did not reveal orthopedic problems commonly found in PTHS, such as scoliosis, deep thoracic kyphosis, or funnel chest. Among the examined most used orthopedic aids such as shoe insoles (*n* = 3), lower limb orthoses (*n* = 5), orthopedic shoes (*n* = 4), walkers (*n* = 3), standing frames (*n* = 2), and stabilization suit (*n* = 1).

None of the examined children required surgical correction of postural defects. The presence of specific orthopedic problems in the studied group is illustrated in Table 5.

In the examined children with PTHS, other problems were identified, such as bruxism and dental caries (*n* = 4), with one child requiring dental treatment under general anesthesia according to the caregivers’ questionnaire.

Sensory integration process disorders were found in seven children, with five exhibiting tactile hypersensitivity. It is noteworthy that intellectual disability was observed in all children who underwent testing, with three having a moderate intellectual disability, one having a significant degree, and one having profound intellectual disability. In the open-ended question regarding the observed progress of the child, each parent indicated that developmental advancement was slow but noticeable. Two caregivers observed significant progress in therapy and the child’s functioning in society after achieving walking ability.

None of the examined girls displayed urogenital organ defects. In the examined boys, both cases presented with a small penis.

The remaining clinical problems reported in the study are presented in Table 6.

### 3.3. Functioning of Families with PTHS

The examined families with PTHS consisted, on average, of 4 members (min. = 3, max. = 6, Standard Deviation (SD) = 0.99). The median income of the surveyed families was PLN 7500 (min. = PLN 3000–5000, max. = over PLN 10,000, SD = 0.99), with 50% of the respondents considering their financial situation sufficient to cover the child’s treatment. The median expenditure on rehabilitation and medical care ranges from PLN 1000 to 2000 (min. up to PLN 500, max. = PLN 2000–3000, SD = 1.19). In only one family, both parents were employed, while in the remaining families, in six out of seven cases, the child’s illness led to the mother resigning from work.

In all families, the primary caregiver for the child was the mother, with one family additionally receiving assistance from a caregiver. Three out of eight families reported in the survey that they did not accept their child’s illness. More than 50% of families observed a deterioration in family relationships due to the child’s illness, citing a lack of time for parents to pursue hobbies and a lack of opportunities for child-free relaxation. Families dedicated on average about 8 h per week to therapy and rehabilitation of their child (min. = 5 h, max. = 10 h, SD = 2.59). The median distance to the rehabilitation centers is 30 km (min. < 10 km, max. > 100 km, SD = 1.19).

Six out of eight families received assistance from foundations and associations, with subsidies for equipment for the child ranging from PLN 2000 to 6000 annually. The primary therapeutic methods applied for the children in the study were the NDT-Bobath method (*n* = 7) and sensory integration therapy (*n* = 7). Additionally, parents mentioned the Vojta method, animal-assisted therapy, craniosacral therapy, hippotherapy, the Tomatis method, and the Krakow method. Six out of eight parents considered their overall knowledge about the disease unsatisfactory. Parents mostly expanded their understanding through the Internet (*n* = 8), by exchanging experiences with other parents (*n* = 8), during medical consultations (*n* = 3), and by participating in conferences/meetings (*n* = 3). None of the responders were members of an association dedicated to PTHS, but all families are part of a Facebook community uniting affected families.

Three out of eight parents considered the level of medical care unsatisfactory, noting a lack or slight improvement in the quality of life after the diagnosis and the beginning of the treatment. Three out of eight caregivers regarded the medical care of their children as average, and only two respondents considered it as fairly good. The most neglected areas of medical care were lack of knowledge about the disease among medical staff (*n* = 7), coordination of medical care (*n* = 4), access to medical care (*n* = 4), professionalism of medical staff (*n* = 2), and cost-effectiveness (*n* = 2).

### 3.4. Assessment of PedsQL

Only three out of eight parents scored an overall QoL score above 50 points in the study. The worst-performing dimension was related to the ability to focus and dedicate time to daily household activities, as well as the dimension concerning worries (M = 43.14; SD = 20.17) related to therapy and the child’s health. The best-performing dimension was cognitive functioning (M = 70.63; SD = 21.95), focusing on concentration and memory skills. The average QoL for families with PTHS in the studied group was 53.82. The results of the PedsQL for parents are presented in Table 7.

Parents suggested the following solutions to improve the QoL: increasing general knowledge about the disease (*n* = 4), rise in the number of educational and rehabilitation facilities for children with disabilities (due to distance from their place of residence) (*n* = 2), increasing funds allocated for treatment (*n* = 3), more time dedicated to specialists (*n* = 2), and assistance in coordinating the child’s treatment and rehabilitation (*n* = 1).

### 3.5. Assessment of QL-Disability

The study, through which parents assessed the QoL of their children, revealed the greatest limitations in the section related to daily life, where questions primarily addressed independence, functioning capabilities, and the ability to influence daily activities. The best results were obtained in the section concerning the child’s ability and freedom to express positive emotions. The average value of QoL for children with PTHS, in the studied group, was 67.70. The results of the Quality-of-Life Inventory—Disability are presented in Table 8.

### 3.6. The COVID-19 Pandemic and Families with PTHS

In the study group, an attempt was made to assess the situation of families with PTHS in the context of the COVID-19 pandemic. The study indicated that all parents of children with PTHS were vaccinated against COVID-19. Among children with PTHS, only two were vaccinated, with three parents expressing a willingness to vaccinate their child once vaccinations become available for their age group (at the time of the study, vaccinations were available for children from the age of five). The remaining parents cited the perceived lack of necessity for vaccination and complications they had observed in themselves as post-vaccination reactions as reasons for not vaccinating their child. Fifty percent of the children participating in the study had COVID-19, but none required hospitalization due to the disease. In one child, parents observed increased nervousness and more frequent abdominal pain as a complication after the illness. Four out of eight families considered their mental well-being worse compared to the time before the pandemic, three families regarded it as similar, and one family judged it as better. According to most parents (six out of seven parents, one respondent marked “not applicable”), the quality of their children’s education has remained at a similar level as before the pandemic. Slightly worse was the availability of doctors (six out of eight respondents considered it as similar, while two as worse) and physiotherapy centers (five out of eight parents stated that the availability was comparable, and three respondents judged it has worsened).

## 4. Discussion

Pitt–Hopkins syndrome (PTHS) is a severe developmental disorder affecting the psychomotor abilities of the child, thereby impacting various aspects of the child’s life, hindering social interactions, and preventing the attainment of independence and autonomy in adult life [1,8]. This study examined a group of eight families to describe the genotype and phenotype of children with PTHS living in Poland. Simultaneously, the study aimed to assess the quality of life of these families.

In all of the studied children, changes in the *TCF4* gene were identified, but the specific mutations varied in each case. The variants described in the study do not align with those previously reported by researchers such as Giurgea et al. and Mary et al. [5,13]. The latest literature reports depict patients exhibiting a phenotype typical for PTHS but with variations in the *SOX11* variant (causing Coffin–Siris syndrome). However, none of the patients included in the study had a similar mutation [16]. It should also be noted that there are reports of the possibility of mutations typical for PTHS with a clinically mild course, as described by Aldeeri in a family where alterations in the *TCF4* gene were detected in three relatives [17]. Genetic testing of all studied children revealed de novo mutations, indicating that parents did not show signs of gene mutations. These findings are consistent with existing literature [1,3,4,5].

Each child exhibited a phenotype typical for PTHS, including a broad nose, full cheeks, and a distinct Cupid’s bow, partially aligning with data from a 2016 study describing the PTHS phenotype and the frequency of specific features in a group of 47 subjects [8]. In contrast to the mentioned article, we did not report a protruding lower part of the face (mainly the jaw) in the studied group. However, this element of the phenotype was not described as being a typical PTHS feature in the latest scientific reports [4,12]. All children met the cardinal criterion of presenting at least three phenotypic features typical for PTHS, following diagnostic guidelines [4,12].

Concerning neurodevelopmental disorders, the most common problems included abnormal speech development, diminished response to pain, and hypotonia. Only one child had epilepsy (drug-resistant), constituting a small percentage within the cohort compared to the literature data [18]. For those children for whom it was possible (due to age), intelligence quotient (IQ) tests verified intellectual disability. Additionally, the majority of the participants experienced challenges in mobility and walked with a wide base. These characteristics are consistent with the literature [3,4,8,12]. No hearing impairment, which could explain communication problems, was observed.

Gastroenterological issues, particularly constipation, were a common problem among children with Pitt–Hopkins syndrome. In light of published reports on the potential for fatal complications arising from gastrointestinal disorders, particular attention should be paid to this issue [19]. Although constipation has been described in the literature, its frequency was lower than observed in the studied group [8,20]. Pharmacotherapy was introduced for some children facing this issue. Reports suggest the potential use of manual (derived from osteopathic techniques) treatment for constipation in PTHS children [21]. Gastroesophageal reflux disease occurs less frequently in the studied group compared to the literature data [20]. Apnea, typical for PTHS, was infrequent in the studied group (two out of eight cases). On the other hand, hyperventilation was found in six out of eight subjects. No correlations were observed between episodes of apnea or hyperventilation and epileptic seizures, as only one child in the studied group had epilepsy [4,12]. Although individuals with PTHS are at an increased risk of developing Hirschsprung’s disease, it was not observed in any of the children [4].

Orthopedic issues primarily involved deformities requiring orthotic supplies for the feet (flat, clubfoot, cavus foot). No posture-related spinal deformities were identified in the studied group, likely due to the young age of the subjects. Deformities such as scoliosis or exaggerated kyphosis of the thoracic spine usually develop during rapid growth, which had not yet occurred in any of the studied young children. Due to reduced muscle tone and increased risk associated with the genetic defect, regular musculoskeletal examinations are advisable for these children [4,12]. Other frequently occurring issues included cold hands (though none of the parents reported in the questionnaire disturbances in maintaining a constant body temperature, which may occur in PTHS), drooling, and sensory integration disorders, which were addressed through therapy in all children for whom it was diagnosed. No genitourinary defects were found in examined girls, although such defects occur in females with PTHS. Moreover, boys in the study group presented with a small penis, which was convergent with the literature [4].

Results from QCP regarding the QoL of PTHS families showed that in half of the surveyed families, the disease contributed to deteriorated relationships and giving up hobbies. Half of the respondents had no time for leisure without their child. Moreover, in six out of eight cases, the child’s illness forced the mother to resign from employment. These are challenges arising from the chronic condition of the child that parents must cope with. The necessity of rehabilitation, visits to specialists, and increased need for care due to intellectual disability led mothers to resign from work, which significantly affected their relationship with family members and their children [22]. The median income of the families involved in the study amounted to PLN 7500, which exceeds the average earnings in Poland at that time (according to data from the Central Statistical Office, the min. wage in the year 2022 amounted to PLN 3010 while the average wage was PLN 6346) [23]. However, it should be noted that the average salary is calculated per capita, whereas the data in the study encompass the budget of the entire family, with only one parent employed in most cases.

The quality of medical care was deemed quite good by only two respondents. Participating parents drew attention to aspects they believed needed improvement. A considerable majority, seven out of eight respondents, expressed dissatisfaction with the level of knowledge about the disease among medical personnel. Other significant problems included the coordination of healthcare (largely related to the need to combine therapy and treatment in multiple centers) and access to medical care (long queues, few centers, often significantly distant from the place of residence). The challenges of parents were multifaceted and resulted from systemic problems (e.g., long queues to specialist doctors). Establishing an association of parents of children with Pitt–Hopkins syndrome, aiming to disseminate knowledge about the syndrome, increase awareness of the needs of PTHS children, and influence decision-makers to improve the functioning of medical services, could be helpful.

The average QoL of families of children with PTHS was low, reaching 53.82 out of 100 possible points. This level was similar to the group of patients with Down syndrome where it was 54.4/100 points and in the group of patients with Rett syndrome—50.94/100 [24,25]. However, it is lower than observed among families caring for children at home under long-term care conditions, as described in a 2004 study that applied the same scale, with an average score of 62.49 [26]. The QoL for children with PTHS was determined using the Quality-of-Life Inventory—Disability version for parents. The calculated average quality of life for the studied group was 67.70 out of 100, with parents identifying major issues in the child’s independence in daily functioning, possibly stemming from communication difficulties and intellectual disability. The average QoL determined using this questionnaire for groups of children with disorders such as autism spectrum disorder, cerebral palsy, Down syndrome, and Rett syndrome was 69, slightly higher than the group of children with PTHS who participated in the study [27]. Addressing this issue may involve further development in speech therapy and exploring alternative communication methods with the child to actively engage them in the family’s daily life.

In the QCP, respondents were also asked about the impact of the COVID-19 pandemic on their functioning. As revealed, in contrast to studies [28] conducted on a group of patients with rare diseases and metabolic disorders in Spain, the majority of patients in the examined group did not notice a significant difference in the functioning of educational or medical centers. This may be attributed to the fact that the study was conducted in 2021 when most centers had already returned to standard operations, maintaining minimal restrictions such as mask-wearing and hand sanitization. A concerning aspect is that some parents express reluctance to vaccinate their children against COVID-19, despite the literature describing no contraindications to vaccinations in PTHS [4,12]. It should be the attending physicians’ responsibility to raise awareness among parents regarding this matter.

## 5. Conclusions

The study facilitated the collection of the phenotype and genotype data of children affected by Pitt–Hopkins syndrome (PTHS) in Poland. Phenotypic changes were found to align with the descriptions presented in the literature. Genetic findings contribute to the expansion of genotype data on PTHS.

The quality of life of families of children with PTHS was observed to be lower than in published works, particularly when compared to families caring for children requiring constant care in home conditions. The quality of life of children with PTHS appeared similar to the levels observed in the other genetic syndromes.

The period of the COVID-19 pandemic did not significantly impact the functioning of families with children affected by PTHS.

This study serves as an initial step in the dialogue between parental organizations and healthcare providers concerning the needs of families of children affected by PTHS. This dialogue can contribute to improving their quality of life and enhancing the quality of healthcare in rare diseases.

## Figures and Tables

**Table 1:** Genetic test result, type of performed test, and age of child at the time of diagnosis in children with Pitt–Hopkins Syndrome.

Patients Number	*TCF4* Gene Variant	Type of Test	Age at Diagnosis
1	c.2045G>A, p.Arg682Gln	NGS, panel ID genes	2 years
2	c.2126A>C, p.Lys709Thr	Targeted *TCF4* sequencing	2 years
3	c.1711C>T, pArg571Cys	NGS, WES	8 years
4	c.1739G>C, p.Arg580Pro	Targeted *TCF4* sequencing	2 years
5	c.583_593del, p.Asn195AlafsTer15	Targeted *TCF4* sequencing	4 years
6	c.1741G>C, p.Val581Leu	NGS, WES	3 years
7	c.1071delT, p.Ala357fs	NGS, panel ID genes	Data not available
8	c.2044C>T, p.Arg682Trp	Targeted *TCF4* sequencing	6 years

NGS—next-generation sequencing, ID—intellectual disability, WES—Whole Exome Sequencing.

**Table 2:** Occurrence of dysmorphic features in the study group.

Phenotype	Patient								Total
	1	2	3	4	5	6	7	8	
Narrow forehead	+	+	−	+	−	−	+	+	5
Thin outer part of eyebrows	+	−	−	−	+	+	+	+	5
Broad nose	+	+	+	+	−	+	+	+	7
Nostril wings turned outward	−	+	+	+	+	−	−	+	5
Full cheeks	+	−	+	+	+	+	−	+	6
Large lips	+	+	+	−	−	−	+	+	5
Full mouth	+	+	+	−	+	+	−	−	5
Distinct Cupid’s bow	+	+	−	+	+	+	−	+	6
Thick and slightly curved earlobes	−	+	−	+	−	+	+	+	5
Small and slender hands	+	+	−	−	−	−	+	+	4

“+”—present, “−”—absent.

**Table 3:** Occurrence of neurodevelopmental problems in the study group.

Neurodevelopmental Problem		Patient								Total
		1	2	3	4	5	6	7	8	
Epilepsy		+	−	−	+	−	−	−	−	2
Wide-based gait		−	−	+	+	+	+	−	+	5
Hypotonia		+	+	+	+	+	+	−	−	6
Sleep disorders		−	−	+	+	−	−	−	+	3
Vision disorders	Strabismus	+	−	−	+	+	−	+	+	5
	Astigmatism	+	+	+	+	−	−	+	−	5
	Myopia	+	−	−	−	−	−	+	+	3
	Blocked tear ducts	+	−	−	−	−	−	−	−	1
	Nystagmus	+	−	−	−	−	−	−	+	2
	Hyperopia	−	−	+	−	−	−	−	−	1
	Cortical visual impairment	−	−	−	+	−	−	−	−	1
Diminished response to pain		+	−	+	+	+	+	+	+	7
Abnormal speech development		+	+	+	+	+	+	+	+	8
Hearing impairment		−	−	−	−	−	−	−	−	0

“+”—present, “−”—absent.

**Table 4:** Occurrence of gastrointestinal problems in the study group.

Gastrointestinal Problem		Patient								Total
		1	2	3	4	5	6	7	8	
Wide-spaced teeth		+	+	+	+	+	−	+	+	7
Problems with food intake	Choking on solid food	+	−	−	−	−	+	−	−	2
	Choking on liquid food	+	−	−	+	−	+	−	+	4
	Choking episodes during meals	−	+	+	−	+	−	−	+	4
	Frequent belching	+	−	−	−	+	−	−	+	3
	Eating at specific time and place	−	+	−	−	+	−	−	−	2
	Greedy eating	−	−	+	−	−	−	−	−	1
	Selective eating	−	−	−	+	+	−	−	−	2
	Lack of biting skills	+	+	−	−	+	−	−	−	3
	Large appetite	−	−	+	−	−	−	−	+	2
Apnea		+	−	−	+	−	−	−	−	2
Hyperventilation		+	+	+	+	−	−	+	+	6
Frequent upper respiratory tract infections		+	−	−	+	+	+	−	+	5
Reflux		−	−	−	−	+	−	−	−	1
Hirschsprung’s disease		−	−	−	−	−	−	−	−	0
Constipation		+	+	+	+	+	−	+	+	7

“+”—present, “−”—absent.

**Table 5:** Occurrence of orthopedic problems in the study group.

Orthopedic Problem	Patient								Total
	1	2	3	4	5	6	7	8	
Lack of independent walking ability	+	+	−	+	−	−	+	−	4
Clubfoot	+	−	+	+	−	−	−	+	4
Flat feet	−	−	+	−	−	−	−	−	1
Cavus foot	−	−	−	−	−	−	+	−	1
Overlapping toes	+	−	−	−	+	−	−	−	2
Limited finger mobility	−	−	−	−	+	−	−	−	1
Scoliosis	−	−	−	−	−	−	−	−	0
Deep thoracic kyphosis	−	−	−	−	−	−	−	−	0
Funnel chest	−	−	−	−	−	−	−	−	0
Reduced knee joint mobility	−	−	−	−	+	−	−	−	1

“+”—present, “−”—absent.

**Table 6:** Other clinical problems in the study group.

Other Clinical Problems	Patient								Total
	1	2	3	4	5	6	7	8	
Bruxism (teeth grinding)	−	−	−	−	+	+	+	+	4
Dental caries	−	−	−	−	+	+	+	+	4
Sensory integration disorders	+	−	+	+	+	+	+	+	7
Intellectual disability	n.as.	n.as.	+	+	+	n.as.	+	+	5
Cold limbs	+	−	+	+	+	+	+	+	7
Dilated pupils	+	−	+	−	+	−	−	−	3
Drooling	+	+	+	+	−	+	+	+	7
Small penis	n.ap.	+	n.ap.	+	n.ap.	n.ap.	n.ap.	n.ap.	2

“+”—present, “−”—absent, “n.as”—not assessed (child too young), “n.ap”—not applicable.

**Table 7:** Quality of Life using the PedsQL questionnaire.

Problem with…	Result of the PedsQL Questionnaire					
	*N*	M	Me	Min	Max	SD
Physical functioning	8	57.29	54.17	37.00	100.00	19.00
Emotional functioning	8	54.38	50.00	35.00	100.00	20.08
Social functioning	8	51.56	59.38	0.00	100.00	33.03
Cognitive functioning	8	70.63	67.50	45.00	100.00	21.95
Communication	8	51.04	45.83	16.67	100.00	26.14
Worries	8	43.13	45.00	10.00	75.00	20.17
Daily activities	8	30.21	25.00	0.00	100.00	29.86
Family relationships	8	60.63	62.50	30.00	100.00	24.41
Total score	8	53.82	48.61	36.11	96.53	19.21

*N*—number of respondents, M—mean, Me—median, Min—minimum value, Max—maximum value, SD—standard deviation.

**Table 8:** Results of the QOL study using the Quality-of-Life Inventory—Disability.

Category	Results of the Quality-of-Life Study Using the Quality-of-Life Inventory—Disability					
	*N*	M	Me	Min	Max	SD
Health and well-being	8	75.00	78.13	50.00	93.8	16.02
Positive feelings	8	86.72	93.75	62.50	100.0	14.34
Negative feelings	8	64.29	60.71	39.29	85.7	15.15
Family and friends	8	68.30	67.86	50.00	82.1	10.53
Outdoor activities	8	67.50	65.00	45.00	100.0	22.04
Daily life	8	44.38	42.50	15.00	80.0	20.78
Total score	8	67.70	67.50	54.49	81.8	9.28

*N*—number of respondents, M—mean, Me—median, Min—minimum value, Max—maximum value, SD—standard deviation.

## Data Availability

The data that support the findings of this study are available from the corresponding author, upon reasonable request.
